# Physical activity and breast cancer in the Multiethnic Cohort Study

**DOI:** 10.21203/rs.3.rs-11177770/v1

**Published:** 2026-10-26

**Authors:** Jasmeet K. Gill, Chloe Holm, Song-Yi Park, Yurii Shvetsov, Christopher Haiman, Lynne R. Wilkens, Loïc LeMarchand, Gertraud Maskarinec

**Affiliations:** California State University; University of Hawai’i Cancer Center, University of Hawai’i; University of Hawai’i Cancer Center, University of Hawai’i; University of Hawai’i Cancer Center, University of Hawai’i; University of Southern California; University of Hawai’i Cancer Center, University of Hawai’i; University of Hawai’i Cancer Center, University of Hawai’i; University of Hawai’i Cancer Center, University of Hawai’i

## Abstract

**Purpose:**

Breast cancer remains the most common cancer among women and risk varies by race/ethnicity. Physical activity has been shown to reduce risk by 10–21% in White women but this reduction has not been established in other races/ethnicities. This study explores the association between physical activity and breast cancer risk in a large cohort of multiethnic women.

**Methods:**

We analyzed data from 93,137 African American, White, Japanese American, Latina, and Native Hawaiian women who participated in the Hawaii-California Multiethnic Cohort Study. Data on physical activity (hours and METs) was based on a self-administered questionnaire at baseline (1993–1996) and a 10-year follow-up questionnaire (2003–2007). Multivariate Cox proportional hazards models provided estimates of hazard ratios (HRs) and 95% confidence intervals (CIs).

**Results:**

We observed an inverse association between breast cancer risk and METs of total activity per day (HR = 0.92, 95% CI: 0.87–0.98, P_trend_=0.01) but not for total hours of moderate/vigorous activity per day (HR = 0.95, 95% CI: 0.89–1.02, P_trend_ = 0.08) when comparing the highest quartile to the lowest in all women. After stratification, Latina women in the highest quartile of METs of total activity per day had a statistically significant reduction in breast cancer risk (HR = 0.84, 95% CI: 0.72–0.98, P_trend_=0.05) compared to the lowest quartile. For others, HRs were below one for the highest METs quartile; there was no evidence of heterogeneity across race/ethnicity (p = 0.76).

**Conclusion:**

This research confirms an overall protective association between physical activity and breast cancer risk, and suggests protection across races/ethnicities.

## INTRODUCTION

Physical activity is hypothesized to affect breast cancer initiation and/or promotion in several ways. It modifies circulating levels of sex hormones (1, 2), growth factors (2), C-reactive protein (3) and improves immune function (4). Continuous physical activity has been shown to sustain global DNA methylation, which is thought to reduce the development of breast cancer (5). Physical activity decreases endogenous estrogen in postmenopausal women by reducing adipose tissue (6), which is a source of estrogen biosynthesis; and a Mendelian randomization study suggests that physical activity decreases risk of breast cancer irrespective of adiposity (7).

There is a plethora of epidemiologic research supporting the inverse association of physical activity and breast cancer. A meta-analysis of 58 case-control and cohort studies estimated a 21% reduction (RR = 0.79, 95% confidence interval (CI): 0.74–0.84) in risk of breast cancer among postmenopausal women with high levels of moderate to vigorous physical activity (8). When the authors restricted analysis to prospective studies only, the risk was attenuated (RR = 0.90, 95% CI: 0.85–0.95) but still statistically significant. Other meta-analyses conducted with data from prospective studies confirmed a statistically significant reduction of breast cancer with physical activity (9, 10) and a statistically significant increase in breast cancer incidence among women who engaged in sedentary work compared to active women (11).

The bulk of the prospective research on physical activity and breast cancer is from studies of White women. To date, there are few large prospective studies of other races/ethnicities. Results from the Black Women’s Health Study, with 1,364 incident cases of breast cancer, were consistent with previous research (12). The authors observed a 26% decrease in risk of breast cancer (95% CI: 0.57–0.96) when they compared the most active to the least active women. Two prospective studies in Asian populations produced mixed results. A Japanese study analyzing 207 incident breast cancer cases observed an HR of 0.45 (95% CI: 0.25–0.78) when they compared the most to the least physically active Japanese women (13); however, another Japanese study with 652 cases observed no association with METs of leisure-time physical activity and breast cancer (14). A Chinese study of 717 incident cases of breast cancer observed no consistent, statistically significant association with physical activity (15).

In this report, we examine data from the large Multiethnic Cohort (MEC) study for possible associations between physical activity and incidence of breast cancer in five racial/ethnic groups in the US: Whites, African Americans, Japanese Americans, Latinas and Native Hawaiians.

## MATERIALS AND METHODS

### Study population

Details of the MEC were described previously (16). In brief, data were collected between 1993–1996 using a 26-page self-administered mail questionnaire sent to residents of Hawaii and California, mainly of Los Angeles County. A follow-up questionnaire in 2003–2007 was sent to all women known to be alive at the time. The relevant Institutional Review Boards approved the study and return of the questionnaire was considered informed consent. Subjects were identified through drivers’ license records in both locations; in addition, voter registration records were used in Hawaii and Health Care Financing Administration files in California. African Americans, Whites, Japanese Americans, Latinas and Native Hawaiians were the primary targets for recruitment, but a small number of persons of other race/ethnicities were also enrolled in the study. Participation in the cohort was limited to people between the ages of 45–75 years at cohort entry, except for Native Hawaiians who were recruited at 42 years and older. A total of 118,913 women completed the survey. After excluding women who did not belong to one of the five major ethnic groups and women who had a prior history of breast cancer 104,046 women remained. We also excluded 1,809 women with missing data on BMI, and 9,100 with missing physical activity information; leaving 93,137 women available for this analysis.

### Assessment of physical activity

The questionnaires included demographic information, body weight and height, and reproductive information, and dietary history (16). The physical activity questions ascertained time spent moving (participating in activity), sleeping, and sitting and were designed to reflect behavior over an average 24-hour period, the year before enrollment in the cohort. Movement was recorded as participation in moderate activities, vigorous work, and strenuous sports. Strenuous sports were defined as jogging, bicycling on hills, tennis, racquetball, swimming laps, or aerobics; vigorous work was defined as moving heavy furniture, loading or unloading trucks, shoveling, weight lifting, or equivalent manual labor; and moderate activity was defined as housework, brisk walking, golfing, bowling, bicycling on level ground, or gardening. Participants recorded length of activity by choosing one of eight categories: never, 1/2–1, 2–3, 4–6, 7–10, 11–20, 21–30, and ≥ 31 hours per week. Sleep duration, including naps, was measured using six categories: ≤5, 6, 7, 8, 9, and ≥ 10 hours of sleep per day. Sitting was defined by sitting activities (watching TV, reading, playing cards, sewing) and sitting in daily routines (in car or bus; at work and at meals). Participants recorded length of sitting by choosing one of seven categories: never, < 1, 1–2, 3–4, 5–6, 7–10, and ≥ 11 hours per day. Hours of total physical activity was computed for the baseline and 2003–2007 follow-up questionnaires.

Hours in each activity level per day was calculated using the midpoint of the categories: 0, 0.75/7, 2.5/7, 5/7, 8.5/7, 15/7, 25.5/7, and 31/7. Light physical activity was calculated by subtracting the total time spent in all activities (sleeping, sitting, or in moderate and vigorous activity) from 24 hours. The metabolic equivalents (METs) for a 24-hour day were created using the following formula: (hours in sleep×0.91 + hours in sitting×1.0 + hours in light activity×2.4 + hours in moderate activities×4.0 + hours in vigorous activity×7.2)/24. Hours of moderate and vigorous activity per week were calculated as the sum of hours in moderate activities and hours in vigorous activity. The MEC physical activity questionnaire was validated against a measure of total energy expenditure based on doubly labeled water in 230 adults (17), with correlation between the objective and self-reported MET values being r = 0.29.

### Case identification and follow-up

The follow-up time started at the date of entry into the cohort, defined as questionnaire completion or the date of the 45th birthday for the few MEC members younger than 45 years at baseline. Follow-up ended at the earliest of the following dates: 1) breast cancer diagnosis, 2) death, or 3) last date of follow-up for cancer incidence. Breast cancer was the event of interest and incident cases were identified through linkages with the Los Angeles County Cancer Surveillance Program, the State of California Cancer Registry, and the Hawaii Tumor Registry. All three are members of the Surveillance, Epidemiology, and End Results program supported by the National Cancer Institute. Information on deaths was obtained from the Vital Statistics offices in both states and the National Death Index. Case ascertainment was complete through December 31, 2021. All breast cancer cases (in-situ and invasive) were identified for the analysis

### Statistical analysis

We used Cox proportional hazards models with age as the time metric to estimate hazard ratios (HRs) and 95% confidence intervals (95% CIs) while adjusting for covariates. Quartiles of the physical activity variables were based on distributions among all women. All models were adjusted for race/ethnicity as a strata variable and age at cohort entry as a covariate. Multivariate models included the following covariates: family history of breast cancer (none, mother/sister/daughter diagnosed with breast cancer), education (≤ 12 years, > 12 years), body mass index (BMI: <20, 20 – < 25, 25 –< 30, ≥ 30 kg/m^2^), alcohol intake (number of drinks per day), smoking history (never, former, current), age at menarche (≤ 12, 13–14, ≥ 15 years), age at first full-term pregnancy (nulliparous, ≤ 20, 21–30, ≥ 31 years), number of children for parous women (1, 2–3, ≥ 4), age at and type of menopause (natural: age < 50, ≥ 50 years; surgical: age < 50, ≥ 50 years), and use of hormone replacement therapy (none, past, current estrogen only, current estrogen and progesterone). These variables were chosen because they are considered established risk factors for breast cancer or because they are associated with risk of breast cancer in the MEC. The exposures of physical activity, BMI and menopause status were repeated on the baseline survey and the follow up survey in 2003–2007 and were modeled as time varying covariates. For women without a follow-up survey, the baseline values were used for their entire follow-up, while for women with a repeat survey prior to the age at the end of follow-up, the 2003–2007 survey values were used starting at the age of the repeat survey. Trend tests were conducted by modeling an ordinal variable reflecting values for each quartile of physical activity. We also performed stratified analyses by race/ethnicity and BMI categories. Tests for interaction were performed by using the Wald test, jointly comparing all interaction terms of physical activity and the subgroup (18).

## RESULTS

Among 93,137 African American, White, Japanese American, Latina, and Native Hawaiian women, 8,667 breast cancer cases were observed during a mean follow-up time of 21 years. We observed a greater proportion of Latina, postmenopausal, parous (with 4 or more children) women in the highest quartile of METs of total activity compared to the lowest quartile (Table 1). Women in the highest quartile were more likely to have a normal BMI and to have never used tobacco.

We analyzed physical activity with two measures: hours of moderate/vigorous physical activity per day and METs of total activity per day. Hours of moderate/vigorous activity per day did not show a statistically significant association with risk of breast cancer, HR = 0.95 (95% CI: 0.89–1.02, Table 2) when comparing the highest quartile to the lowest quartile (P_trend_ = 0.08).

We did observe a statistically significant association between METs of total activity per day and risk of breast cancer, HR = 0.92 (95% CI: 0.87–0.98, Table 2), when comparing the highest quartile to the lowest quartile. For each additional quartile of METs, the risk of breast cancer decreased, P_trend_=0.01. When stratified by BMI categories, we observed no statistically significant association between quartiles of METs of total physical activity per day and risk of breast cancer (See Supplementary Table 1S).

Hazard ratios for women in the highest quartile of METs of total physical activity remained below one for each of the 5 racial/ethnic groups and we observed no evidence of an interaction (P_heterogeneity_ = 0.76, Table 3). The HR was 0.97 (95% CI: 0.84–1.12) for African American women, 0.97 (95% CI: 0.81–1.16) for Native Hawaiian women, 0.93 (95% CI: 0.83–1.04) for Japanese American women, and 0.92 (95% CI: 0.81–1.05) for White women when comparing the highest quartile of METs per day of total physical activity to the lowest quartile. Interestingly, Latina women in the highest quartile of METs had a statistically significant reduction in breast cancer risk, HR = 0.84 (95% CI: 0.72–0.98) compared to those in the lowest quartile and there was a statistically significant trend in reduction of breast cancer risk with increasing METs of physical activity (P_trend_=0.05).

## DISCUSSION

In this large, multiethnic cohort, physical activity, as measured by METs of total activity but not the daily hours of moderate/vigorous activity, was associated with a reduction in breast cancer risk for all women. We observed no statistically significant associations across levels of BMI. When analyzed by racial/ethnic groups, Latina women in the highest quartile of total METs day of physical activity showed a 16% reduction in risk of breast cancer (95%CI: 0.72–0.98) and the HRs for the remaining four racial/ethnic groups suggested reductions in risk of breast cancer, although they were not statistically significant. This could reflect race/ethnic differences in where the women were active (work-related versus recreation) and/or differences in intensity versus amount of activity. Of the five racial/ethnic groups, Latinas spent the least amount of time sitting per day and had the most METs of total activity. They sat an average of 2 hours at work (s.d.=2.82) and had an average of 7.13 hours of total sitting activity per day (s.d.=3.79, Supplemental Table 2S). Their average total activity per day was 1.64 METs, s.d.=0.28).

Our findings confirm the results of published meta-analyses of prospective research since they also found a protective effect of physical activity on risk of breast cancer (8-10). Pizot et al. used data from 38 prospective studies, for a total of 116,304 women with breast cancer and estimated a 12% reduction (95% CI: 0.85–0.90) in breast cancer risk when comparing women in the highest category to the lowest category of physical activity. Wu et al. examined data from 31 prospective studies, resulting in 63,786 women with breast cancer eligible for analysis. They also estimated a 12% reduction (95% CI: 0.85–0.91) in risk of breast cancer when comparing women in the highest category to the lowest category of physical activity. More recent studies confirmed the protective effect of physical activity on breast cancer to varying degrees. The UK Biobank prospective cohort study observed a 13% reduction in postmenopausal breast cancer (95% CI: 0.78–0.98) when they compared the highest quartile of physical activity MET-hours per week to the lowest (19). The Malmö Diet and Cancer study reported an even greater reduction in risk, HR = 0.77 (95% CI: 0.66–0.90), for women with high levels of physical activity at baseline compared to low physical activity (20). Lastly, a Canadian prospective study also observed reductions in risk of breast cancer ranging from 15–25%, but the estimates did not gain statistical significance. Their data produced an HR = 0.75 (95% CI: 0.56–1.00) for recreational physical activity and an HR = 0.85 (95% CI: 0.64–1.14) for total physical activity when comparing the highest quartile of MET-hours per week to the lowest (21).

Our analysis of 8,667 incident cases of breast cancer supports the protective effects of physical activity, but our estimates show a more modest reduction of 8% in risk of breast cancer (95% CI: 0.87–0.98).

Few prospective studies have been conducted in non-whites. Two prospective studies of Japanese women yielded conflicting results. One study observed an HR = 0.85, 95% CI: 0.51–1.40, when comparing women who reported exercising more than 3 hours per week to women who seldom or never exercised. However, the risk reduction was statistically significant when they measured physical activity as a combination of ≥ 1 hour of walking per day and ≥ 1 hour of exercise per week (HR = 0.45, 95% CI: 0.25–0.78) (13). The other study examined daily total physical activity and found no statistically significant association when comparing the highest to the lowest tertile of METs per day (RR = 0.90, 95% CI: 0.68–1.19, P_trend_ = 0.48) (14). Our results of Japanese American women in the highest quartile of METs of total activity per day are similar in magnitude and significance (HR = 0.93, 95% CI: 0.83–1.04, P_trend_ = 0.18).

The Black Women’s Health Study, a large prospective study of African American women in the US, gathered data on walking and on vigorous exercise for their participants. Their analysis of 1,364 cases of invasive breast cancer yielded a statistically significant reduction in breast cancer when comparing ≥ 7 hours of vigorous physical activity to < 1 hour per week (IRR = 0.74, 95% CI: 0.57–0.96, P_trend_ =0.05) (12). Women with ≥ 45 MET-hours of vigorous exercise and brisk walking showed a statistically significant reduction (IRR = 0.72, 95% CI, 0.57–0.90) compared to women with none. The researchers observed no statistically significant reduction when analyzing brisk walking alone (IRR = 0.98, 95% CI: .077–1.23, for ≥ 7 hours compared to < 1 hour per week). In contrast, our analysis of African American women in the highest quartile of METs of total activity per day had an HR = 0.97 (95% CI: 0.84–1.12). The differences in physical activity measures may contribute to the discordance in results; they measured vigorous physical activity while we examined total physical activity. However, our analysis of moderate/vigorous activity (hours per day), produced a hazard ratio (HR = 0.95) similar to that of total physical activity (METs per day), HR = 0.92. This may reflect a true attenuated effect of physical activity on the risk of breast cancer in our sample or it may reflect differences in physical activity intensity of the samples.

Many challenges remain in better understanding the magnitude, the biological pathways, and the lifecourse timing of physical activity in relation to breast cancer (22). One challenge is errors in self-reported physical activity data. Recent research has attempted to better capture physical activity by directly measuring it. For example, the U.K. Biobank study used accelerometers to measure physical activity and to compare the levels against the self-report questionnaire data from baseline (23). They observed a reduction in risk of all cancers known to be related to physical activity (HR = 0.69, 95%CI: 0.55–0.86) when comparing the accelerometry measured median daily amount of vigorous intermittent physical activity to no vigorous activity. In the U.S., a research group used prospective data from the Women’s Health Initiative Objective Physical Activity and Cardiovascular Health Study and the Women’s Health Study to analyze accelerometer data among post-menopausal women (24). They observed a reduction in breast cancer risk (HR = 0.80, 95% CI: 0.64–0.99) when comparing the highest to the lowest tertile of vector-magnitude activity. However, this association was not statistically significant after adjustment for BMI as physical activity may act indirectly through its effect on body weight. In stratified analysis by race, no statistically significant associations were seen when comparing the highest to the lowest tertile of vector-magnitude activity; the HR = 0.87 (95% CI: 0.61–1.24) for Black women and HR = 0.94 (95% CI: 0.51–1.76) for Hispanic women (24).

Strengths of our study include the prospective design, the large study population of five racial/ethnic groups and the comprehensive baseline questionnaire with a follow-up. Limitations include potential residual confounding and lack of information on physical activity intensity level by type of activity. Additionally, although we conducted a validation study of the physical activity questions, the correlation with doubly labelled water was modest (17).

In sum, our findings confirm an overall inverse association between physical activity and breast cancer and suggest this effect is similar across race/ethnicity. Future research should gather more detailed physical activity data and confirm self-report with accelerometer recordings across race and ethnicity.

## Supplementary Material

This is a list of supplementary files associated with this preprint. Click to download.

- SUPPLEMENTARYTABLES.docx
- Table3.docx

## Figures and Tables

**Table 1:** Baseline characteristics of participants by physical activity

		Total Activity (METs/day)			
		≤ 1.40	1.41- ≤1.60	1.61- ≤1.76	≥ 1.77
No. Participants		23347	23264	23242	23284
No. Breast cancer cases		2314	2229	2200	1924
Age at Cohort Entry (yr.)*		57.05 (8.47)	59.19 (8.83)	60.44 (8.73)	59.67 (8.62)
Moderate/vigorous physical activity (hours/day)*		0.63 (0.50)	0.88 (0.82)	1.04 (1.03)	1.92 (1.87)
Ethnicity, (%)	African American	21.51	18.79	17.21	15.90
	Native Hawaiian	8.39	7.47	6.47	8.21
	Japanese American	28.38	29.32	30.58	25.19
	Latina	16.89	17.88	21.28	26.18
	White	24.83	26.55	24.46	24.53
Education, (%)	12 years or less	37.22	40.41	46.79	50.74
	12 + years	62.02	58.76	52.34	48.11
Family History of Breast Cancer, (%)		10.48	10.81	10.68	10.35
BMI (kg/m^2^)*		27.55 (6.49)	26.52 (5.79)	26.01 (5.40)	25.92 (5.39)
BMI Category, (%)	Underweight	2.22	2.64	2.91	2.87
	Normal weight	37.96	43.80	46.40	47.28
	Overweight	31.41	31.41	31.50	30.99
	Obese	28.41	22.15	19.19	18.86
Smoking Status, (%)	Never	50.35	53.60	57.24	57.89
	Former	30.56	30.87	28.56	27.68
	Current	18.02	14.40	12.90	12.75
Age at Menarche, (%)	≤ 12 years old	52.31	50.71	48.87	47.05
	13–14 years old	36.03	37.38	38.06	38.58
	≥ 15 years old	10.63	10.90	12.03	13.17
Number of Children, (%)	None	15.61	13.24	11.82	10.25
	1 child	12.55	12.12	10.70	10.32
	2–3 children	44.44	44.98	45.31	42.24
	4 or more children	26.58	28.63	31.22	36.05
Age at First Birth, (%)	Nulliparous	15.75	13.39	12.01	10.43
	≤ 20 years old	28.32	26.90	27.00	30.97
	21–30 years old	48.09	50.77	52.03	49.97
	≥ 31 years old	5.81	6.77	6.85	6.39
Hormone Therapy Use, (%)	Never	52.71	50.48	51.10	53.13
	Past	15.29	15.75	16.40	15.77
	Current Estrogen, No Progesterone	13.31	13.97	13.69	12.67
	Current Estrogen with Progesterone	16.18	16.93	15.60	15.02
Menopausal Status, (%)	Premenopausal	18.40	14.77	12.91	13.12
	Natural menopause < 50 yrs	22.15	22.55	23.43	23.59
	Natural menopause ≥ 50 yrs	22.72	25.53	26.63	25.98
	Surgical menopause < 50 yrs	34.00	34.13	33.66	34.09
	Surgical menopause ≥ 50 yrs	2.73	3.02	3.36	3.23
Alcohol Use, (%)	< 1 drink/month	60.48	61.73	63.41	62.93
	1 drink/day	26.71	26.22	25.24	24.10
	> 1 drink/day	9.05	9.08	8.20	8.73

* Mean (standard deviation)

**Table 2:** Association between physical activity and breast cancer risk in the MEC, 1993–2021

Model	Quartile	Cases	Person Years	HR* (CI)
Moderate/vigorous activity (hours/day)	Q1 (≤ 0.36)	1494	373288	1.00 (ref)
	Q2 (0.37 – ≤0.71)	2133	477874	0.99 (0.93, 1.06)
	Q3 (0.72 – ≤1.21)	2872	656404	0.96 (0.90, 1.02)
	Q4 (≥ 1.22)	2168	525937	0.95 (0.89, 1.02)
	*P for trend*			*0.08*
Total activity (METs/day)	Q1 (≤ 1.40)	2314	513702	1.00 (ref)
	Q2 (1.41-≤1.60)	2229	504486	1.01 (0.95, 1.07)
	Q3 (1.61-≤1.76)	2200	500333	0.99 (0.93, 1.05)
	Q4 (≥ 1.77)	1924	514981	0.92 (0.87, 0.98)
	*P for trend*			*0.01*

* Adjusted by Cox proportional hazards regression for age at cohort entry, race/ethnicity, BMI, education, age at menarche, menopause age and type, number of children, hormonal therapy use, smoking status, alcohol consumption, and family history of breast cancer. Tests for trend were conducted using ordinal categories for each quartile of activity.

## Data Availability

The data that support the findings of this study are available from the Multiethnic Cohort Study upon reasonable request and with permission of the Multiethnic Cohort Study through the online data request system (https://uhcancercenter.org/research/mec/data-sharing).

## References

[1] Friedenreich CM, Woolcott CG, McTiernan A (2010) Alberta physical activity and breast cancer prevention trial: sex hormone changes in a year-long exercise intervention among postmenopausal women. J Clin oncology: official J Am Soc Clin Oncol 28:1458–1466 10.1200/JCO.2009.24.9557 PMC2849767 20159820

[2] Rinaldi S, Kaaks R, Friedenreich CM (2014) Physical activity, sex steroid, and growth factor concentrations in pre- and post-menopausal women: a cross-sectional study within the EPIC cohort. Cancer causes control: CCC 25:111–124 10.1007/s10552-013-0314-x 24173534

[3] Lou MWC, Drummond AE, Swain CTV (2023) Linking Physical Activity to Breast Cancer via Inflammation, Part 2: The Effect of Inflammation on Breast Cancer Risk. Cancer epidemiology, biomarkers & prevention: a publication of the American Association for Cancer Research, cosponsored by the American Society of Preventive Oncology. 32:597–605 10.1158/1055-9965.EPI-22-0929 PMC10150245 36867866

[4] Shephard RJ, Rhind S, Shek PN (1995) The impact of exercise on the immune system: NK cells, interleukins 1 and 2, and related responses. Exerc Sport Sci Rev 23:215–241 7556351

[5] Boyne DJ, O'Sullivan DE, Olij BF, King WD, Friedenreich CM, Brenner DR (2018) Physical Activity, Global DNA Methylation and Breast Cancer Risk: A Systematic Literature Review and Meta-Analysis. Cancer epidemiology, biomarkers & prevention: a publication. of the American Association for Cancer Research, cosponsored by the American Society of Preventive Oncology 10.1158/1055-9965.EPI-18-0175 29991518

[6] Friedenreich CM, Woolcott CG, McTiernan A (2011) Adiposity changes after a 1-year aerobic exercise intervention among postmenopausal women: a randomized controlled trial. Int J Obes (Lond) 35:427–435 10.1038/ijo.2010.147 PMC3061001 20820172

[7] Papadimitriou N, Dimou N, Tsilidis KK (2020) Physical activity and risks of breast and colorectal cancer: a Mendelian randomisation analysis. Nat Commun 11:597 10.1038/s41467-020-14389-8 PMC6992637 32001714

[8] Neilson HK, Farris MS, Stone CR, Vaska MM, Brenner DR, Friedenreich CM (2017) Moderate-vigorous recreational physical activity and breast cancer risk, stratified by menopause status: a systematic review and meta-analysis, vol 24. Menopause (New York, pp 322–344 10.1097/GME.0000000000000745 27779567

[9] Pizot C, Boniol M, Mullie P (2016) Physical activity, hormone replacement therapy and breast cancer risk: A meta-analysis of prospective studies. European journal of cancer (Oxford, England: 1990). 52: 138–54 10.1016/j.ejca.2015.10.063 26687833

[10] Wu Y, Zhang D, Kang S (2013) Physical activity and risk of breast cancer: a meta-analysis of prospective studies. Breast cancer research and treatment. 137: 869–82 10.1007/s10549-012-2396-7 23274845

[11] Lee J, Lee J, Lee DW, Kim HR, Kang MY (2021) Sedentary work and breast cancer risk: A systematic review and meta-analysis. J Occup Health 63:e12239 10.1002/1348-9585.12239 PMC8221371 34161650

[12] Rosenberg L, Palmer JR, Bethea TN, Ban Y, Kipping-Ruane K, Adams-Campbell LL (2014) A prospective study of physical activity and breast cancer incidence in African-American women. Cancer epidemiology, biomarkers & prevention: a publication of the American Association for Cancer Research, cosponsored by the American Society of Preventive Oncology. 23:2522–2531 10.1158/1055-9965.EPI-14-0448 PMC4221421 25103823

[13] Suzuki S, Kojima M, Tokudome S (2008) Effect of physical activity on breast cancer risk: findings of the Japan collaborative cohort study. Cancer epidemiology, biomarkers & prevention: a publication of the American Association for Cancer Research, cosponsored by the American Society of Preventive Oncology. 17:3396–3401 10.1158/1055-9965.EPI-08-0497 19029398

[14] Suzuki R, Iwasaki M, Yamamoto S (2011) Leisure-time physical activity and breast cancer risk defined by estrogen and progesterone receptor status–the Japan Public Health Center-based Prospective Study. Preventive medicine. 52: 227–33 10.1016/j.ypmed.2011.01.016 21295062

[15] Pronk A, Ji BT, Shu XO (2011) Physical activity and breast cancer risk in Chinese women. Br J Cancer 105:1443–1450 10.1038/bjc.2011.370 PMC3241547 21934685

[16] Kolonel LN, Henderson BE, Hankin JH (2000) A multiethnic cohort in Hawaii and Los Angeles: baseline characteristics. Am J Epidemiol 151:346–357 10.1093/oxfordjournals.aje.a010213 PMC4482109 10695593

[17] Wilkens LR, Murphy SP, Jardack P, White KK, Kolonel LN, Arab L (2011) Validation of a physical activity questionnaire against the doubly-labeled water standard among a multiethnic population. FASEB J 25: 978.9–.9.

[18] Therneau TM, Grambsch PM (2000) Modeling survival data: extending the Cox model. Springer, New York

[19] Guo W, Fensom GK, Reeves GK, Key TJ (2020) Physical activity and breast cancer risk: results from the UK Biobank prospective cohort. British journal of cancer10.1038/s41416-019-0700-6 PMC7054300 31919405

[20] Boraka Ö, Klintman M, Rosendahl AH (2022) Physical Activity and Long-Term Risk of Breast Cancer, Associations with Time in Life and Body Composition in the Prospective Malmö Diet and Cancer Study. Cancers (Basel). 14 10.3390/cancers14081960 PMC9025884 35454864

[21] Barberio AM, Friedenreich CM, Lynch BM, Campbell KL, Arora P, Brenner DR (2018) Physical Activity and Cancer Incidence in Alberta's Tomorrow Project: Results from a Prospective Cohort of 26,538 Participants. Cancer epidemiology, biomarkers & prevention: a publication of the American Association for Cancer Research, cosponsored by the American Society of Preventive Oncology. 27:945–954 10.1158/1055-9965.EPI-17-1124 30026295

[22] Yang L, Courneya KS, Friedenreich CM (2024) The Physical Activity and Cancer Control (PACC) framework: update on the evidence, guidelines, and future research priorities. Br J Cancer 131:957–969 10.1038/s41416-024-02748-x PMC11405831 38926526

[23] Stamatakis E, Ahmadi MN, Friedenreich CM (2023) Vigorous Intermittent Lifestyle Physical Activity and Cancer Incidence Among Nonexercising Adults: The UK Biobank Accelerometry Study. JAMA Oncol 9:1255–1259 10.1001/jamaoncol.2023.1830 PMC10375384 37498576

[24] Hyde ET, LaCroix AZ, Evenson KR (2023) Accelerometer-measured physical activity and postmenopausal breast cancer incidence in the Women's Health Accelerometry Collaboration. Cancer 129:1579–1590 10.1002/cncr.34699 PMC10133094 36812131

